# Diaphragmatic hernia during treatment of lung cancer harboring an EGFR mutation

**DOI:** 10.1093/omcr/omab054

**Published:** 2021-07-21

**Authors:** Aya Konno-Yamamoto, Osamu Narumoto, Shota Yamamoto, Miho Yamaguchi, Makoto Motoyoshi, Yuta Inoue, Takeshi Fukami, Atsuhisa Tamura, Hirotoshi Matsui

**Affiliations:** 1 Center for Pulmonary Disease, National Hospital Organization Tokyo Hospital, Kiyose, Tokyo 204-8585, Japan; 2 Department of Thoracic Surgery, National Hospital Organization Tokyo Hospital, Kiyose, Tokyo 204-8585, Japan; 3 Department of Radiology, Tokai University Hachioji Hospital, Tokai University School of Medicine, Hachioji, Tokyo 192-0032, Japan; 4 Department of Gastroenterological Surgery, National Hospital Organization Tokyo Hospital, Kiyose, Tokyo 204-8585, Japan

## Abstract

Epidermal growth factor receptor (EGFR) tyrosine kinase inhibitors (TKIs) are a first-line treatment for patients with nonsmall-cell lung cancer harboring EGFR mutations. We report a 65-year-old Japanese woman with nonsmall-cell lung cancer taking an EGFR-TKI who visited the emergency department with acute nausea and vomiting. Imaging studies demonstrated an incarcerated diaphragmatic hernia. Urgent diagnostic surgery revealed a gap in the diaphragm acting as a hernial orifice, where a metastatic tumor was detected. We consider that regression of the diaphragmatic metastasis by EGFR-TKI therapy resulted in perforation of the diaphragm, causing the diaphragmatic hernia. Gastrointestinal adverse events, e.g. nausea, vomiting and diarrhea, are common during EGFR-TKI treatment. However, this case suggests that in patients with diaphragmatic metastasis, we should consider the rare possibility of diaphragmatic perforation and a subsequent hernia.

## INTRODUCTION

Epidermal growth factor receptor (EGFR) tyrosine kinase inhibitors (TKIs) are first-line therapy for patients with nonsmall-cell lung cancer (NSCLC) harboring EGFR mutations. Compared with platinum-based chemotherapy, EGFR-TKIs sometimes lead to significant tumor regression. There have been a few reports of organ perforation, such as pneumothorax, gastric [1] and duodenal [2] perforations, caused by tumor regression after EGFR-TKI treatment. However, there are no reports of diaphragmatic perforation and secondary hernia. Here, we report a case where tumor regression of diaphragmatic metastasis following EGFR-TKI therapy caused perforation leading to a diaphragmatic hernia (DH).

**Figure 1:**
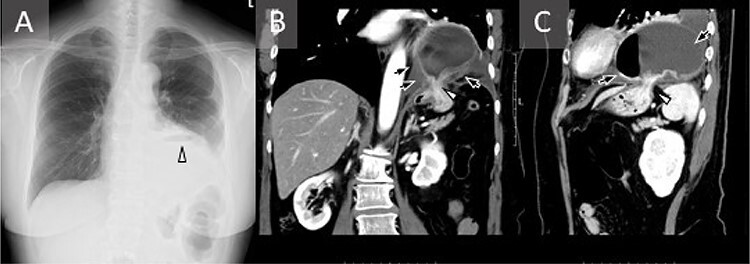
Chest radiography and computed tomography (CT) images obtained **2** days after admission. (A) The gastric bubble is located above the elevated left hemidiaphragm (white arrowhead). (B, C) The contrast-enhanced CT scan shows the hernia (white arrow), with a contrast defect in part of the gastric wall (black arrow).

**Figure 2:**
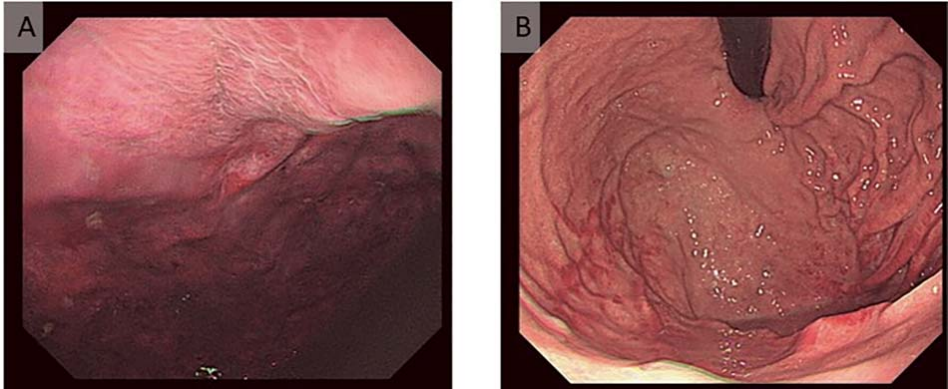
Gastrointestinal endoscopic findings. (A) With the diagnosis of diaphragmatic hernia, color change, erosion and edema were found in the gastric mucosa. We attempted to repair the hernia endoscopically, but without success. (B) One month after urgent surgery, the mucosal color, erosion and edema had significantly improved.

**Figure 3:**
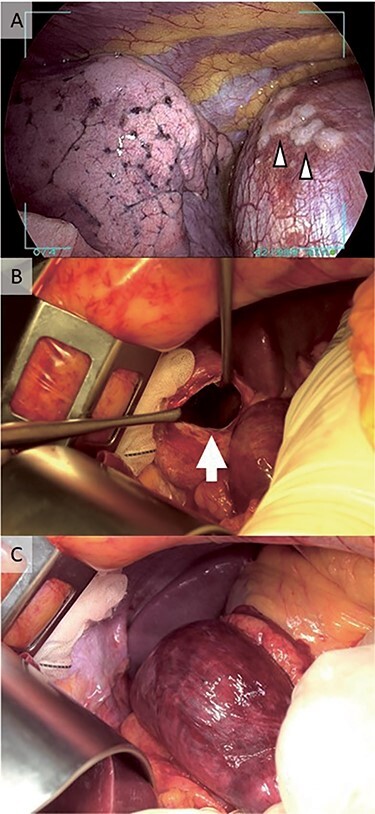
Intraoperative findings. (A) Thoracoscopic view of the diaphragm is shown. White arrowhead shows tumor dissemination. (B) A defect in the diaphragm (white arrow) was detected during laparotomy, located where the tumor had disseminated to the diaphragm. (C) The dark red color of the stomach found during laparotomy suggested disruption of the blood supply.

## CASE REPORT

A 65-year-old Japanese woman presented to the emergency department with acute nausea and vomiting. A year earlier, she was diagnosed with stage IIB (cT1bN1M0) lung adenocarcinoma harboring EGFR exon 19 deletion, which was treated with surgery. During the surgery, unexpected tumor metastases on the pleura and left diaphragm were detected (Fig. 3A), and the surgery was suspended. Soon after, afatinib was administrated as first-line therapy. After 6 months, a complete response of the tumor was achieved.

The patient visited the emergency department with nausea. There was no abnormality on physical examination or abdominal radiography. Infectious colitis or an adverse drug reaction related to the EGFR-TKI was suspected; she underwent intravenous rehydration therapy, which improved her symptoms, and she walked back home. The next day, she presented again with nausea and was admitted to the hospital. Her vital signs were in the normal range. There were no abnormal findings on physical examination and no abdominal pain. Other than a slightly increased white blood cell count (9800/μl), laboratory examinations were normal. Two days after admission, her nausea worsened and we performed chest X-ray and computed tomography (CT). The left diaphragm was elevated according to the chest X-ray (Fig. 1A), and chest CT revealed a DH (Fig. 1B and C). Upper gastrointestinal endoscopy showed an abnormal color change in a gastric mucosal lesion (Fig. 2A). We suspected a strangulated hernia with gastric incarceration and performed urgent laparoscopic surgery. Although the gastric mucosa had turned dark red (Fig. 3C), the color recovered soon after repair of the hernia, and no organ resection was necessary. We observed a defect in the diaphragm (Fig. 3B) where we had previously detected diaphragmatic metastasis. The hole was sutured, and 1 month after the surgery, we confirmed that her gastric mucosal color had improved (Fig. 2B).

## DISCUSSION

Pleural spread in NSCLC is reported in 8–15% patients on baseline imaging [3]. In addition, 3.7% of patients undergoing surgical treatment have detectable pleural dissemination during surgery [4]. Thin-slice CT has a sensitivity of 33–87.5% [5, 6], and positron emission tomography/CT often reports false-negative results [7]. In our case, we could not detect pleural dissemination before surgery. Figure 3A shows pleural dissemination detected during video-assisted thoracoscopic surgery. We found a defect at the same site during treatment of the DH by laparoscopy (Fig. 3B). We suspect that tumor regression caused by the EGFR-TKI resulted in diaphragmatic perforation, and the pressure difference between the thoracic and abdominal cavities resulted in the DH. The intrathoracic pressure is reported to be ~100 cm H_2_O lower than the intra-abdominal pressure during maximal inspiratory effort [8]. In addition, vomiting increases intra-abdominal pressure, which may also have played a role in the development of the DH.

DH is caused by congenital or acquired defects in the diaphragm [9]. Acquired DH is rare and can go unnoticed during the immediate phase without obvious symptoms; this diagnostic delay leads to an overall mortality rate of up to 31% [10]. Since DH does not recover spontaneously, incarcerated organs lead to necrosis and perforation. Therefore, urgent surgery is necessary once a diagnosis of DH is made.

In conclusion, although EGFR-TKIs often cause gastrointestinal symptoms, we should consider the rare possibility of DH in lung cancer patients with potential metastasis to the diaphragm.
